# Supplementary figures and images for: Perforin-2 is essential for intracellular defense of parenchymal cells and phagocytes against pathogenic bacteria
Source: eLife. 2015 Sep 24;4:e06508. doi: 10.7554/eLife.06508 (PMC4626811; doi:10.7554/eLife.06508)

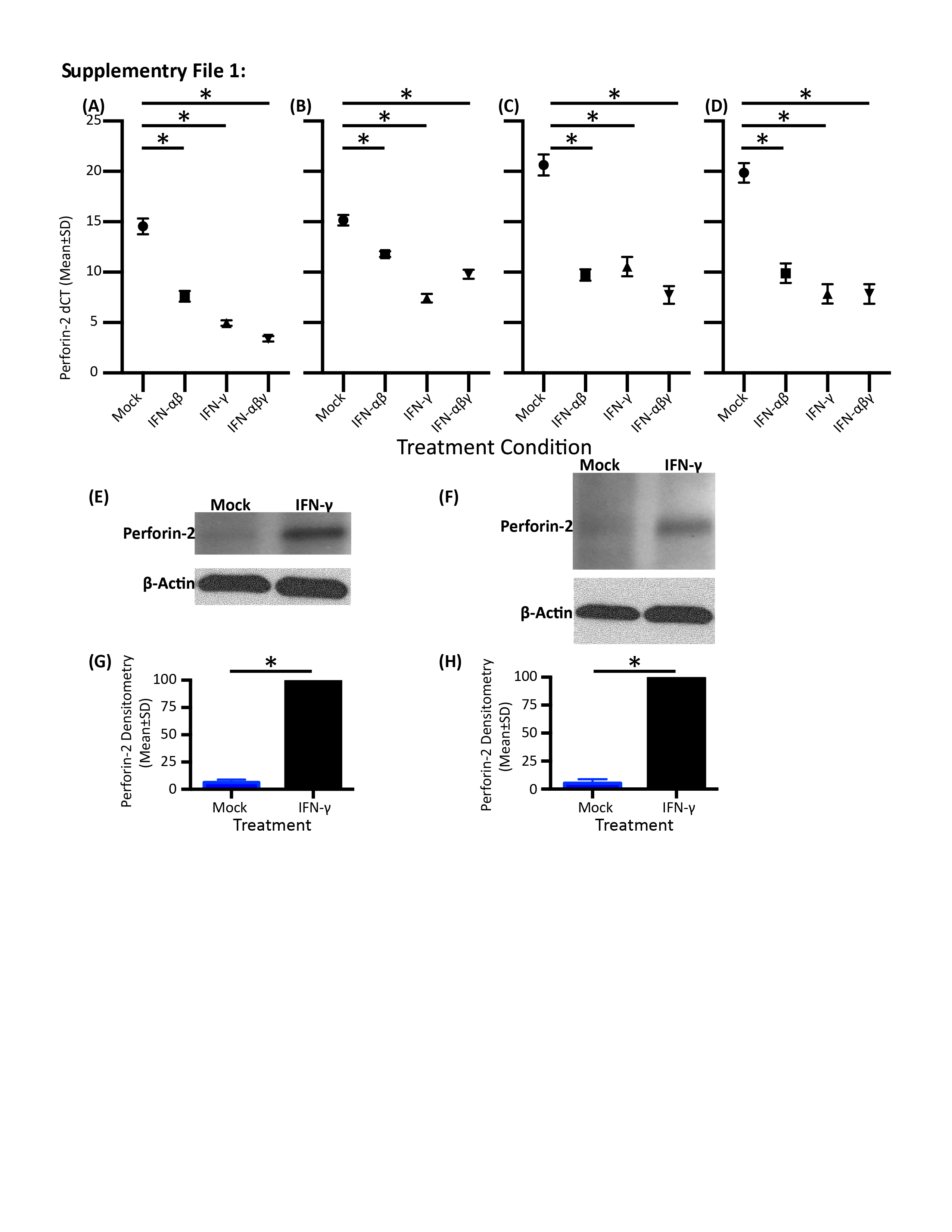

Supplement: Supplementary file 1. — Type I and Type II interferon increase Perforin-2 message in murine non-hematopoietic cell lines. (A–D) Select murine cell lines from Table 1 indicating qPCR delta CT (Perforin-2 normalized to GAPDH) (five experimental replicates) following Type I (Interferon-α, β stimulation), Type II (Interferon-γ stimulation), or both Type I and II (Interferon-αβγ stimulation). (A) Ovarian cancer cell line MOVCAR 5009, (B) Cath.a neuroblastoma cell line, (C) C2C12 myoblast cell line, (D) B16-F10 melanoma cell line. (E–H) Interferon stimulation corresponds with an increase in Perforin-2 protein. (E) Ovarian Cancer MOVCAR 5009 and (F) C2C12 myoblast cell line. Densitometry analysis of five experimental replicates of (G) MOVCAR 5009 or (H) C2C12. (A–D) Statistical analysis was performed with one-way ANOVA with Tukey post-hoc multiple comparisons. (G, H) Statistical analysis was performed with Student's T-test. *p < 0.05. DOI: http://dx.doi.org/10.7554/eLife.06508.032 [file elife06508s001.tif]

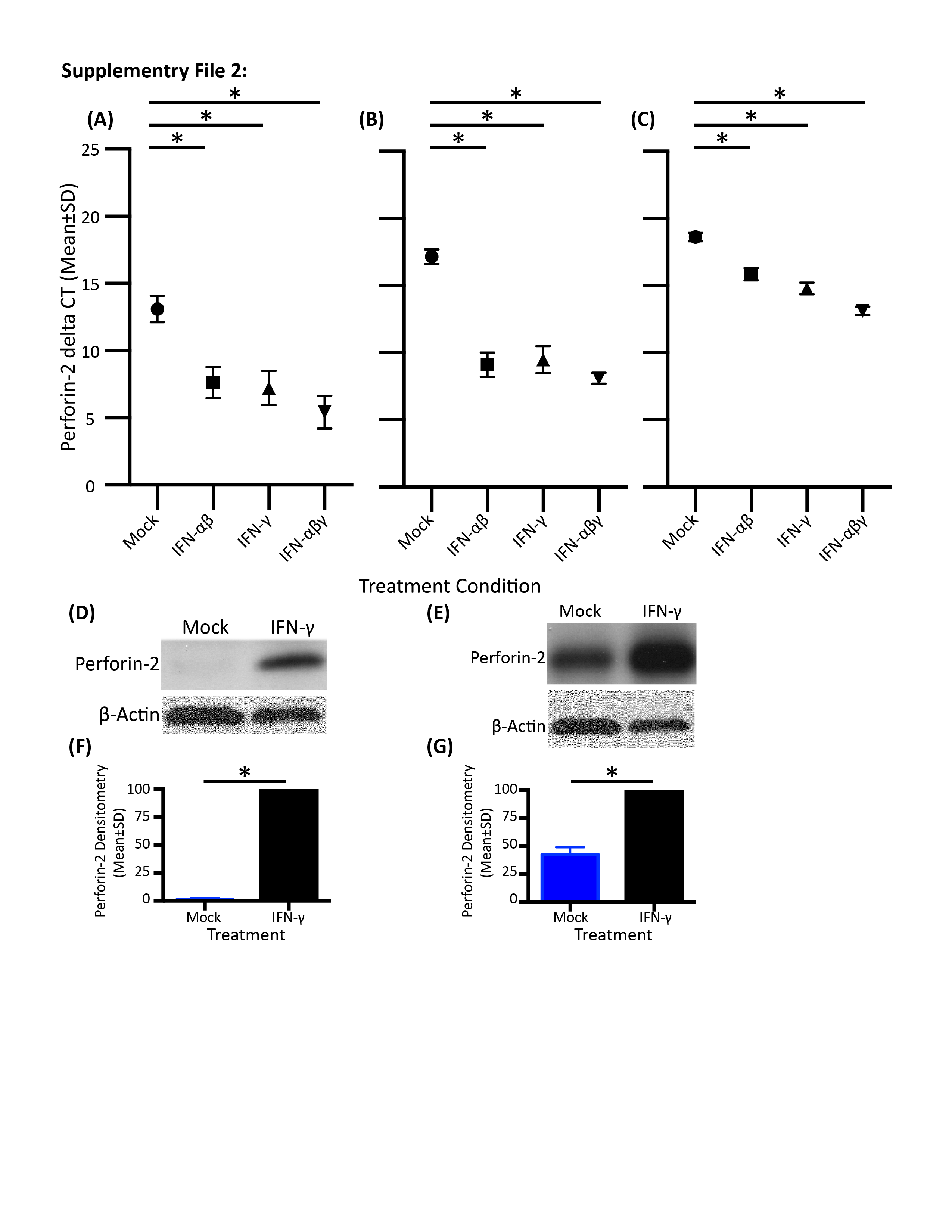

Supplement: Supplementary file 2. — Type I and Type II interferon increase Perforin-2 message in human non-hematopoietic cell lines. Select human cell lines from Table 2 analyzed by qPCR demonstrating delta CT (Perforin-2 normalized to GAPDH) (five experimental replicates) after Type I (Interferon-αβ stimulation), Type II (Interferon-γ stimulation), or both Type I and II (Interferon-αβγ stimulation). (A) Primary HUVEC cells, (B) HEK293 cell line, and (C) MIA-PaCa-2 pancreatic cancer cell line. Interferon stimulation also increased human Perforin-2 protein with (D) MIA-PaCa-2 and (E) HUVEC cell lines. Densitometry analysis of five experimental replicates of (F) MIA-PaCa-2 or (G) HUVEC. (A–C) Statistical analysis was performed with one-way ANOVA with Tukey post-hoc multiple comparisons. (F, G) Statistical analysis was performed with Student's T-test. *p < 0.05. DOI: http://dx.doi.org/10.7554/eLife.06508.033 [file elife06508s002.tif]

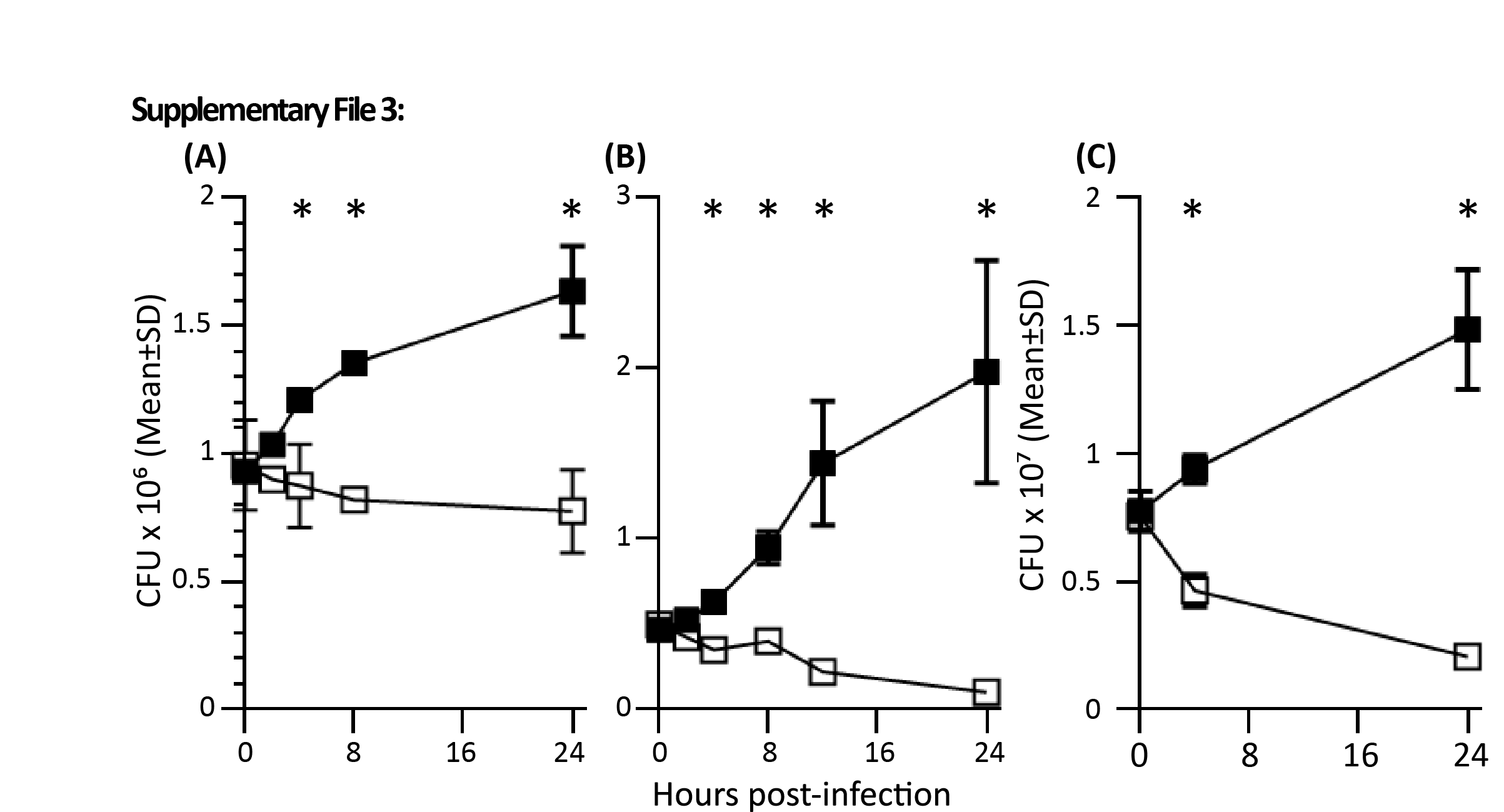

Supplement: Supplementary file 3. — Perforin-2 significantly contributes to intracellular killing in murine non-hematopoietically derived cells. (A–C) One day prior to the experiment, cells were transfected with either a pool of scramble (□) or murine Perforin-2 specific (■) siRNA and 14 hr prior to the experiment induced with IFN-γ. (A) MOVCAR 5009 infected with S. typhimurium, (B) CT26 infected with MRSA, or (C) C2C12 infected with M. smegmatis. The above graphs contain 5 biologic replicates, and are representative of 3 independent experiments. Statistical analysis was performed utilizing multiple T-tests with correction for multiple comparisons using the Holm-Sidak method. *p < 0.05. DOI: http://dx.doi.org/10.7554/eLife.06508.034 [file elife06508s003.tif]

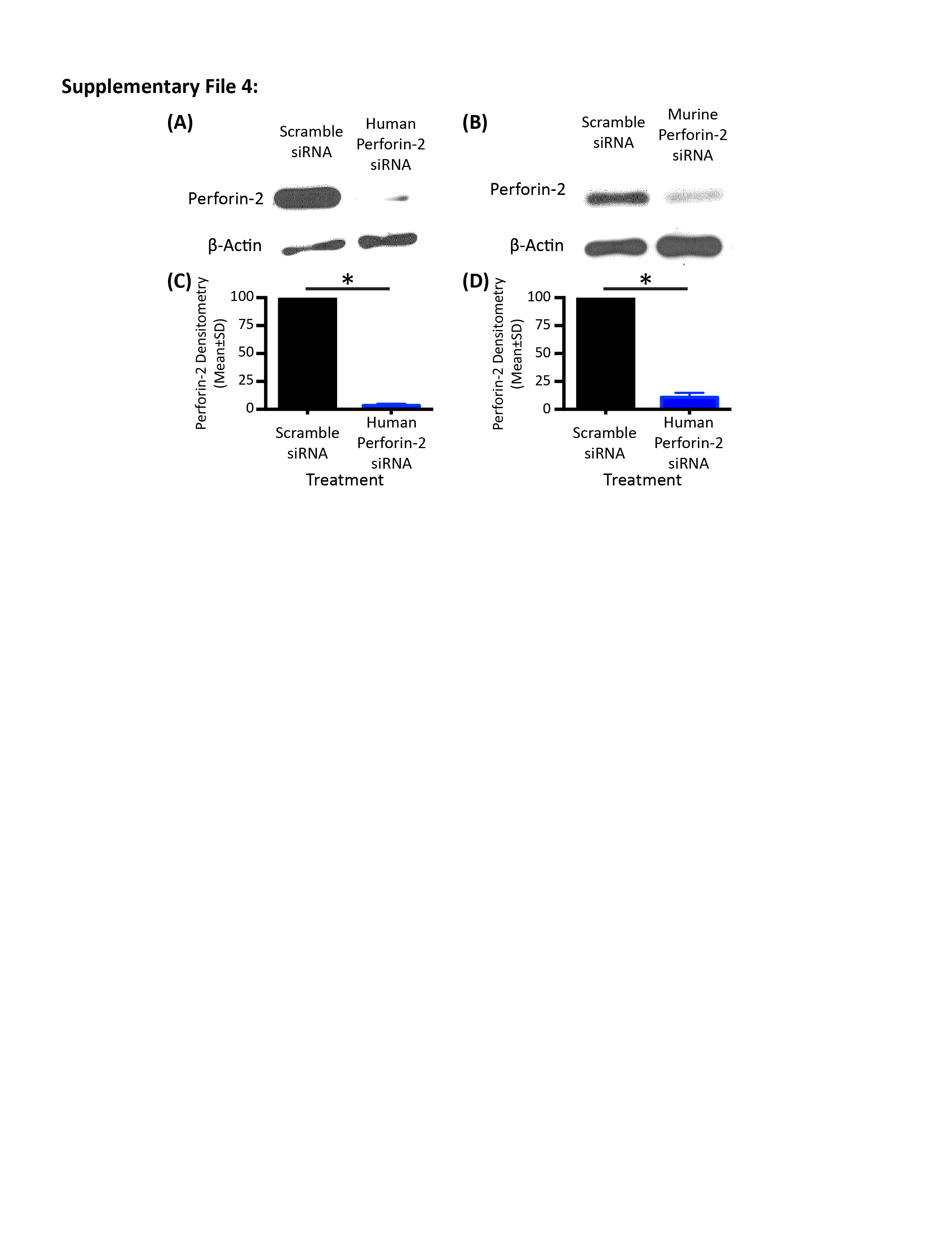

Supplement: Supplementary file 4. — Perforin-2 siRNA knockdown is efficient in both murine and human cells. (A–D) Representative blots from knockdown of selected cells from Table 1, Table 2, and Figure 3. (A, C) Human HUVEC cells representing knockdown of human Perforin-2; (B, D) Murine C2C12 myoblast cell line-demonstrating knockdown of murine Perforin-2. Cells were transfected with either a pool of human or mouse Perforin-2 specific siRNA or scramble siRNA. Cells were induced for 14 hr with IFN-γ and 24 hr post-transfection lysed for protein quantification. Statistical analysis was conducted utilizing Student's T-test. *p < 0.05. DOI: http://dx.doi.org/10.7554/eLife.06508.035 [file elife06508s004.tif]
